# Design of Friction Stir Welding Tool for Avoiding Root Flaws

**DOI:** 10.3390/ma6125870

**Published:** 2013-12-12

**Authors:** Shude Ji, Jingwei Xing, Yumei Yue, Yinan Ma, Liguo Zhang, Shuangsheng Gao

**Affiliations:** Faculty of Aerospace Engineering, Shenyang Aerospace University, Shenyang 110136, China; E-Mails: happyxingjingwei@163.com (J.X.); yueyumei999@yeah.net (Y.Y.); tigerman7@163.com (Y.M.); zlgiast@163.com (L.Z.); gaoshsh@163.com (S.G.)

**Keywords:** friction stir welding, material flow, numerical simulation, root flaws

## Abstract

In order to improve material flow behavior during friction stir welding and avoid root flaws of weld, a tool with a half-screw pin and a tool with a tapered-flute pin are suggested. The effect of flute geometry in tool pins on material flow velocity is investigated by the software ANSYS FLUENT. Numerical simulation results show that high material flow velocity appears near the rotational tool and material flow velocity rapidly decreases with the increase of distance away from the axis of the tool. Maximum material flow velocity by the tool with the tapered-flute pin appears at the beginning position of flute and the velocity decreases with the increase of flow length in flute. From the view of increasing the flow velocity of material near the bottom of the workpiece or in the middle of workpiece, the tool with the half-screw pin and the tool with the tapered-flute pin are both better than the conventional tool.

## 1. Introduction

Friction stir welding (FSW), invented in The Welding Institute, is a solid-state joining technique that has been used successfully in many industries, including aerospace, automotive, railway, and maritime [1,2,3]. Compared to conventional fusion welding, FSW has many advantages, for example, low residual stress, low energy input, non-pollution, high-quality, *etc.* [4].

Material flow behavior is one of the most important key factors to influence the quality of FSW weld, which has led many researchers to investigate material flow by means of experimental methods or numerical simulation methods [5,6,7,8,9,10,11,12]. Seidel *et al.* [5] experimentally examined material flow behavior in FSW using a marker material technique and provided a semi-quantitative view of the material transport in the weld zone. Pourahmad *et al.* [7] showed by X-ray images that material flow of the implanted steel shot in the advancing side is bigger than that in the retreating side. Ji *et al.* [8] by the software FLUENT showed that the tool with one-spiral-flute shoulder is better than the tool with a concentric-circles-flute shoulder. Grujicic *et al.* [9] by coupled Eulerian/Lagrangian computational analysis attained the effects of weld pitch, tool tilt-angle and tool pin size on material flow during the FSW process.

In the practical welding process, the root flaws are the most common defects, which may appear near the bottom of the weld and are bad for the quality of weld. In this manuscript, two kinds of tool pin with special geometry are put forward, and material flow behavior during the FSW process is discussed by using the software ANSYS FLUENT.

## 2. FVM of Friction Stir Welding

### 2.1. The Designed Rotational Tool

During the FSW process, the welding material is in a plastic state. The material must be stirred and then mixed enough to get a sound FSW weld. Therefore, it is necessary for the material to undergo the violent flow. In fact, many defects of FSW related to material flow include cavity, kissing bond, root flaws, and so on [13]. The rotational tool is made up of a shoulder and a rotational pin. During the FSW process, the tip of the rotational pin is inside the welding material in order to avoid contact between the tool and the backing plate and then the damage of the tool or backing plate. Generally speaking, the distance between the tip of pin and the bottom of the welding workpiece is 0.1~0.2 mm [14]. However, the practical distance between the tip of pin and the bottom of the workpiece is longer or shorter than 0.1~0.2 mm, which results in the change of workpiece thickness or the change of plunging depth of tool. The longer distance may result in the appearance of root flaws because of the lack of enough material flow.

**Figure 1:**
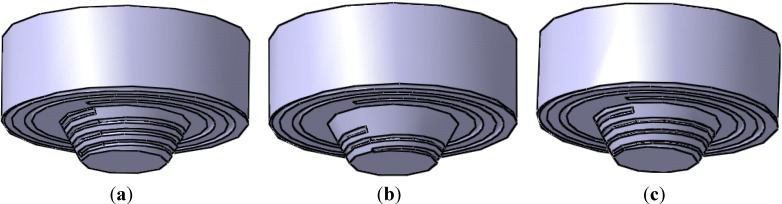
(**a**) The conventional tool used in numerical simulation; (**b**) The tool with half-screw pin used in numerical simulation; (**c**) The tool with tapered-flute pin used in numerical simulation.

How to improve material flow behavior has been attracting researchers’ attention since FSW was invented in 1991. Our project group investigated the effect of shoulder geometry and pin geometry on material flow velocity and then suggested that the flute geometry of the tool is the most important factor to be designed [10,15]. In this manuscript, three kinds of rotational tool are discussed, as shown in Figure 1. Therein, two kinds of rotational tool are designed in order to improve material flow velocity between the tip of tool and the bottom of welding workpiece, as shown in Figure 1b,c.

For the three rotational tools in Figure 1, the dimensions of shoulder, diameters of pin bottom, diameters of pin tip and lengths of pin are all the same. The tool in Figure 1a is called the conventional tool, where width of the flute of the pin is unchanged. The tool in Figure 1b is called the tool with a half-screw pin, where the beginning position of flute along the pin length direction is in the middle of the pin. The tool in Figure 1c is called the tool with the tapered-flute pin, where the width of the flute is changed. In Figure 1a, the diameter of the shoulder is 17 mm and the width of the flute in the shoulder is 0.5 mm. The length of the rotational pin is 2.8 mm, the pin is tapered from 9 mm at the pin bottom to 6 mm at the pin tip and the width of the flute is 0.4 mm. In Figure 1b, the width of the flute of the pin is 0.4 mm. In Figure 1c, the maximum width of the flute of the pin is 0.4 mm and the minimum width is 0.2 mm.

### 2.2. Mesh Generation

**Figure 2:**
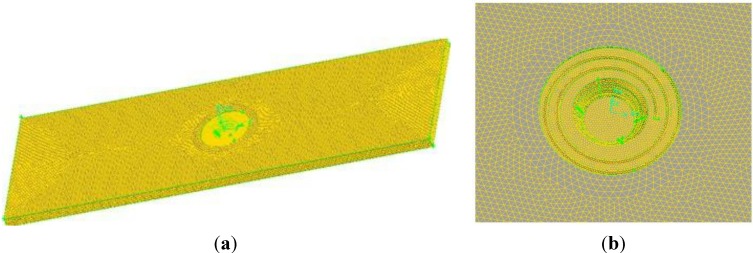
(**a**) Mesh generation of model used in simulation; (**b**) Partial enlarged figure of model.

Figure 2 shows mesh generation of the workpiece used to simulate while the corresponding tool is the tool in Figure 1c. It is well known that material near the rotational tool flows violently during the FSW process, so we are able to use the dimensions of mesh near the rotational tool in order to describe material flow behavior in detail.

### 2.3. Material Parameters

In this manuscript, Ti6Al4V titanium alloy is chosen as investigated subject. The specific heat, the thermal conductivity and the density of Ti6Al4V alloy are 879 J/Kg·K, 15.91 W/m·K and 4.45 g/cm^3^, respectively. The RNG k-ε model is used in this study and the material is supposed to be fused liquid. The viscosity of Ti6Al4V alloy is unchanged and the value is 5.3 mPa·s.

### 2.4. Boundary Condition

During the process of numerical simulation, the plastic material is considered as the fluid, which flows into the computational region by the inlet and flows out of the outlet. The surface of workpiece, the bottom of workpiece, the advancing side, the retreating side, the inlet side and the outlet side are all considered as the moving walls. The speed of moving walls is equal to the welding speed of 75 mm/min and the moving direction is opposite to the welding direction. The elements used to represent the rotational tool are considered as the rotational wall while the rotational velocity of 250 r/min and the rotational direction of the wall are both the same as those of the tool. Moreover, the non-slip boundary condition is used to describe the relation between the material contacting the tool and the rotational tool.

## 3. Results and Discussion

**Figure 3:**
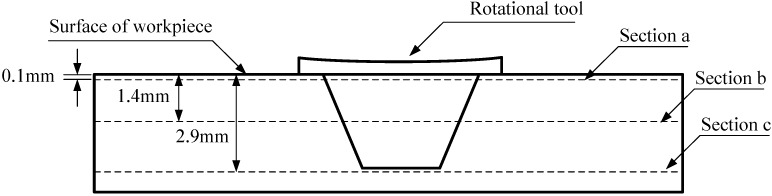
Position of different sections of the workpiece.

In order to research material flow field during FSW, three representative positions are considered: near the surface, in the middle of workpiece, between the pin tip and the bottom of the workpiece. Figure 3 shows three researched sections, which are parallel to the surface of the workpiece and are respectively called sections a, b and c.

### 3.1. Material Flow Behavior of the Tool with Tapered-Flute Pin during FSW

**Figure 4:**
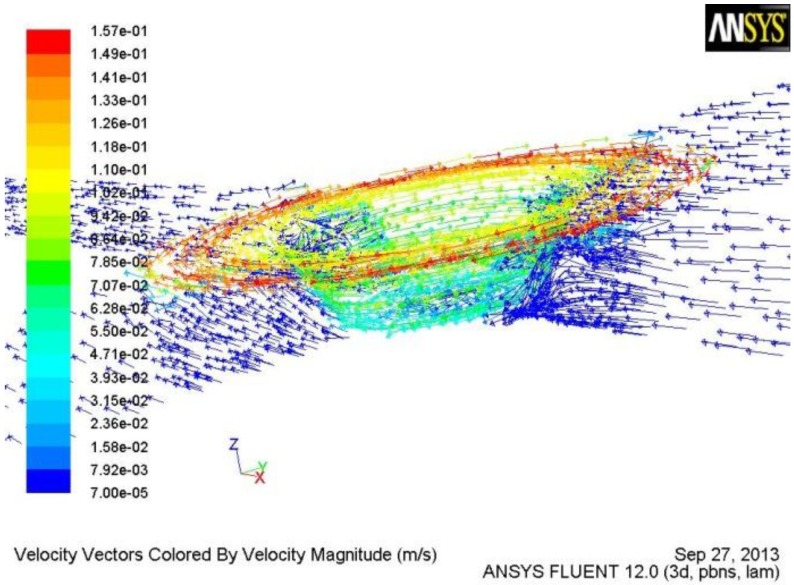
3D material flow field by the tool with the tapered-flute pin.

**Figure 5:**
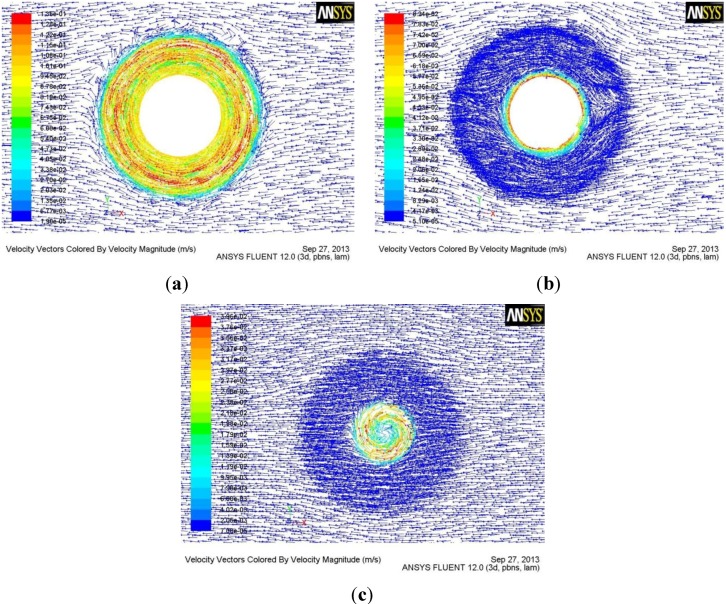
(**a**) Flow field of section a parallel to the workpiece surface; (**b**) Flow field of section b; (**c**) Flow field of section c.

Figure 4 is the 3D material flow field by the tool with the tapered-flute pin of FSW. Figure 5 shows the flow field at the different horizontal sections. The flow velocity of the material in contact with the tool is the same as the linear velocity of the contact position of tool [14,15]. The flow of material in contact with the rotational tool makes the nearby material flow. However, the flow velocity of nearby material is lower than that of the material in contact with the tool in terms of the effect of viscosity of material. Therefore, material flow velocity reduces with the increase of distance away from the rotational axis (Figure 4 and Figure 5). The flow direction of material in contact with the shoulder is the same as the rotational direction of the tool. The flow direction of material in contact with the pin can be divided into two parts. One is the same as the rotational direction of the tool and the other is downward in the vertical direction. Moreover, for material along the vertical direction, the flow velocity and the region under high flow velocity both decrease with the increase of distance away from the workpiece surface, which results from the change of the rotational pin diameter. The flow velocity of the material in the region between the pin tip and the workpiece bottom is relatively small, which may result in root flaws.

### 3.2. Effect of Flute Geometry on Material Flow

**Figure 6:**
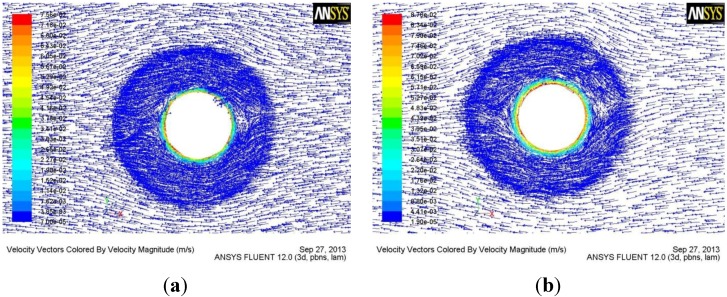
(**a**) Flow field at section b by conventional tool; (**b**) Flow field at section b by the tool with the half-screw pin.

**Figure 7:**
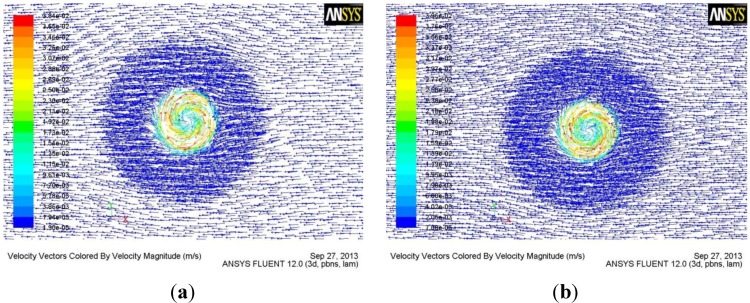
(**a**) Flow field at section c by the conventional tool; (**b**) Flow field at section c by the tool with the half-screw pin.

Figure 6 and Figure 7 respectively show material flow velocity fields by the conventional tool and the tool with the half-screw pin at section b and section c.

From Figure 4 and Figure 5, it is also concluded that the flute of the tool can greatly increase the flow velocity of the material. Therefore, the reasonable design of the flute geometry is very important to the rotational tool of FSW. After fluid flows into the flute, the flow velocity of the material reduces with the increase of flow length. Therefore, the short flute is better than the long flute in terms of increasing the flow velocity of material, which is the reason why the tool with the half-screw pin is designed in order to avoid root flaws. From Figure 6 and Figure 7, it is seen that the tool with half-screw pin can improve the flow velocity of the material not only near the bottom of workpiece but also in the middle of workpiece. Moreover, From Figure 5c and Figure 7, it is seen that material flow velocity in the retreating side is higher than that in the advancing side.

As is reported, the material during FSW process can be considered as the incompressible fluid and the flow behavior follows the continuity law of the fluid [8,13], whose expression is shown in Equation (1):

(1) $$v_{1}A_{1}=v_{2}A_{2}=\text{Constant}$$

where *v* is the flow velocity of the material and *A* is the area of channel cross section. The subscript 1 and 2 mean the cross section 1 and cross section 2, respectively.

From Equation (1), it is known that the flow velocity of material during FSW is inversely proportional to the area of flute. Therefore, a small flute width is beneficial for increasing the flow velocity of the material, which is the reason why the flow velocity of material at section c by the tool with the tapered-flute pin (Figure 5b,c) is higher than that by the conventional tool (Figure 6a and Figure 7a).

## 4. Conclusions

In conclusion, the results of the present study demonstrate that the geometry of the flute of a rotational tool during the friction stir welding process greatly influences the material flow behavior. The tool with half-screw pin and the tool with tapered-flute pin both obviously increase material flow velocity near the bottom of the workpiece and are both beneficial in avoiding root flaws.
